# Discrepancy between predicted and measured respiratory function after thoracoscopic segmentectomy versus lobectomy

**DOI:** 10.1016/j.xjon.2026.102078

**Published:** 2026-08-22

**Authors:** Yukimi Shibuya, Kohei Hashimoto, Kazuharu Suda, Keisei Tachibana, Ryota Tanaka, Haruhiko Kondo

**Affiliations:** Department of Thoracic and Thyroid Surgery, Faculty of Medicine, Kyorin University, Tokyo, Japan

**Figure:**
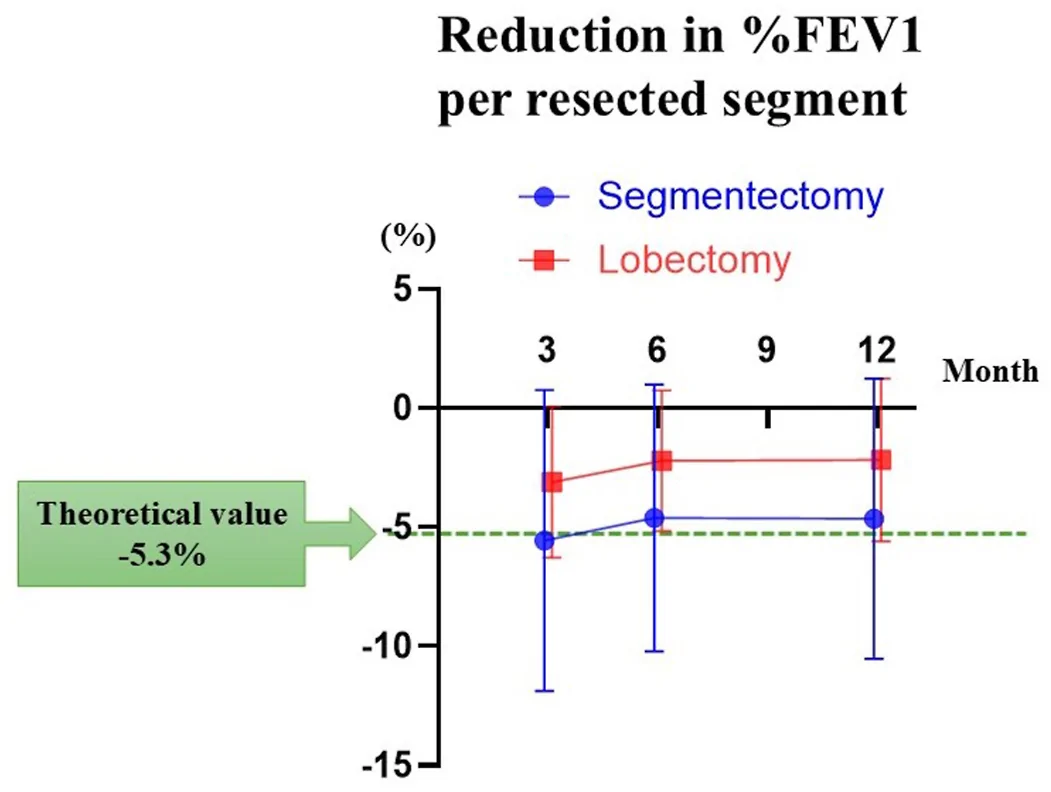
The 19-segment method fits segmentectomy but overestimates %FEV1 loss after lobectomy.

**Central Message**

The conventional segment-counting method accurately predicts postoperative %FEV1 after thoracoscopic segmentectomy but may overestimate functional loss after thoracoscopic lobectomy.

Prediction of respiratory function after lung resection is widely used for preoperative risk stratification. Although the indication for segmentectomy for small peripheral lung cancers evolves rapidly after the randomized controlled trials suggesting the utility of segmentectomy,^1^^,^^2^ it remains unclear whether the conventional prediction method based on segment numbers applies equally to segmentectomy and lobectomy, particularly for thoracoscopic approaches.

## Patients and Methods

The Institutional Review Board or equivalent ethics committee of the Kyorin University approved the study protocol and publication of data (Approval No. 796; Approval Date: May 10, 2021). All patients provided informed written consent for the publication of the study data. Between June 2021 and June 2023, we enrolled 141 consecutive patients who underwent thoracoscopic segmentectomy or lobectomy for suspected lung malignancy and retrospectively analyzed outcomes. Segmentectomy was not limited to patients with impaired pulmonary function but was also selected as an intentional parenchyma-sparing procedure for small peripheral tumors when oncologically appropriate. Predicted postoperative percent forced expiratory volume in 1 second (%FEV1) and percent vital capacity (%VC) were calculated based on 19 segments (proposed by Bolliger and Perruchoud^3^). This method, based on the number of resected pulmonary segments to estimate predicted postoperative function, is widely used and is recommended in both American^4^ and Japanese guidelines. Differences between measured and predicted %FEV1 and %VC at 3, 6, and 12 months and the decrease in measured %FEV1 and %VC per resected segment were compared between the 2 groups using the Mann–Whitney *U* test. Two-group comparisons were performed using the *t* test or Mann–Whitney *U* test, as appropriate. Categorical variables were compared using the Fisher exact test. Statistical analyses were performed using SPSS software (version 28; IBM Corp).

## Results

The segmentectomy group included 34 patients, and the lobectomy group included 107 patients (Table 1). No significant differences were observed in age or surgical side. However, the segmentectomy group had more women and higher preoperative %FEV1. There was no difference in baseline %VC between the groups. Measured and predicted %FEV1 at 3, 6, and 12 months were closely matched after segmentectomy, whereas measured values progressively exceeded predictions after lobectomy, reaching a difference of 10% at 12 months (Table 1). The %FEV1 reduction at 12 months was smaller than the theoretical value (1/19 = 5.3%) for both groups, but significantly smaller in lobectomy (2.2%) than segmentectomy (4.6%, *P* = .02) (Figure 1) (Figure E1 for each replicate). A similar trend was observed in the %VC data.

**Table 1:** Characteristics and difference in percent forced expiratory volume in 1 second of patients undergoing thoracoscopic segmentectomy and lobectomy

Variable	Segmentectomy (n = 34)	Lobectomy (n = 107)	*P* value
Age, y (mean ± SD)	69.5 ± 9.9	69.8 ± 10.8	.87
Male	10 (29.4%)	63 (58.9%)	<.01
Female	24 (70.6%)	44 (41.1%)	
BI	80	430	.07
Right	20 (58.8%)	73 (68.2%)	.31
Left	14 (41.2%)	34 (31.8%)	
Preoperative %FEV1 (mean ± SD)	98.0 ± 19.0	90.7 ± 16.7	.05
Preoperative %VC (mean ± SD)	102.5 ± 13.4	99.6 ± 14.8	.43
Pathology			.74
Primary lung cancer	32	103	
Metastatic lung tumor	1	1	
Inflammatory nodule	1	1	
Others	0	2	
Difference in %FEV1 (measured-predicted)			
3 mo	0.2 ± 7.4	5.6 ± 10.5	<.01
6 mo	−0.0 ± 6.5	9.2 ± 10.5	<.01
12 mo	1.7 ± 7.3	9.7 ± 12.2	<.01
Difference in %VC (measured-predicted)			
3 mo	−1.6 ± 6.5	7.6 ± 10.4	<.01
6 mo	0.1 ± 6.5	11.4 ± 11.1	<.01
12 mo	0.3 ± 6.6	13.5 ± 12.1	<.01

*BI*, Brinkman Index; *%FEV1*, percent forced expiratory volume in 1 second; *%VC*, percent vital capacity.

**Figure 1:**
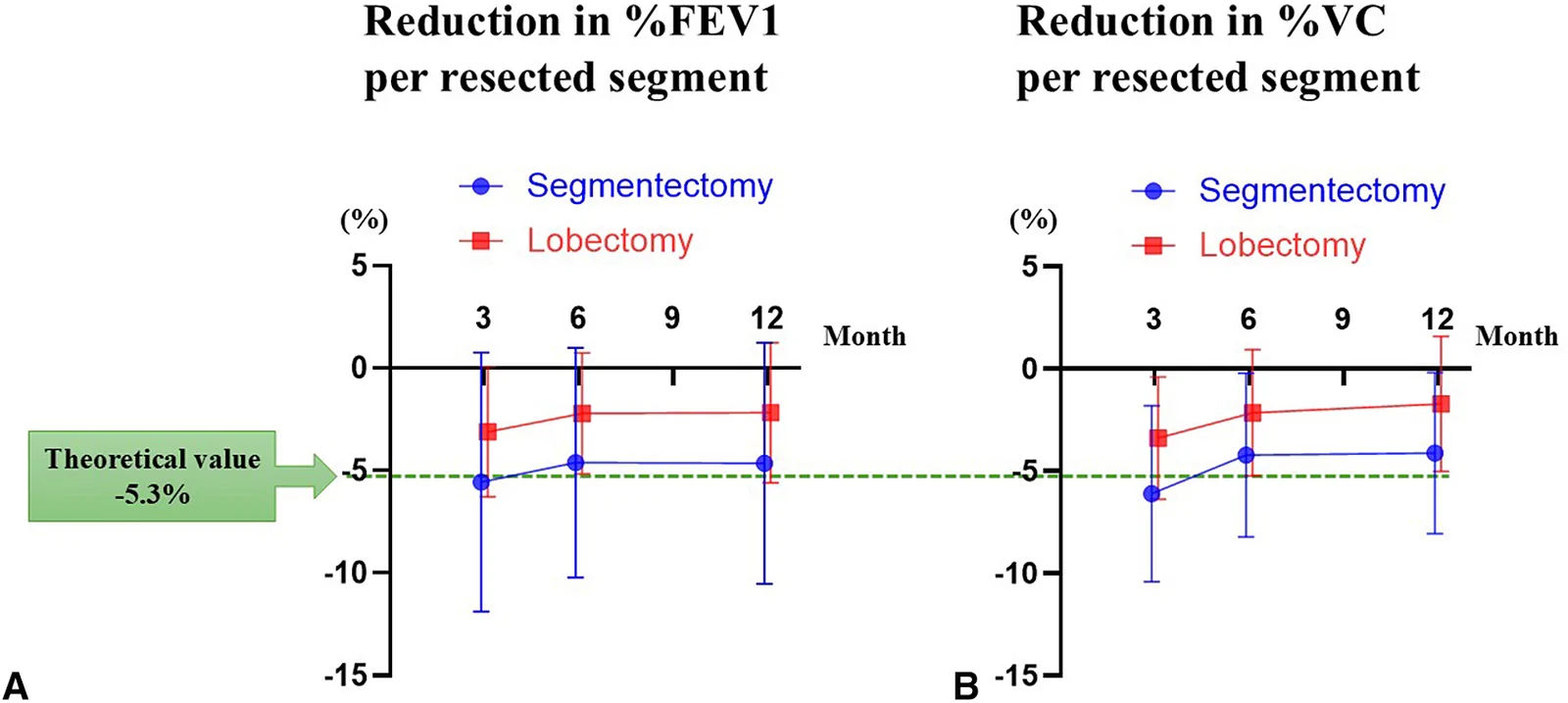
Reduction in %FEV1 (A) and %VC (B) per resected segment at 3, 6, and 12 months after surgery. Patients who underwent segmentectomy (*blue circles*) and lobectomy (*red squares*) are shown. Values are presented as mean ± SD. The *green dashed line* indicates the theoretical value (5.3%) calculated according to Bolliger's method. *%FEV1*, Percent forced expiratory volume in 1 second; *%VC*, percent vital capacity.

The location of tumors is shown in Table 2. There was a statistically significant difference in distribution between the segmentectomy and lobectomy groups. In the segmentectomy group, the number of resected segments was as follows: 1 (N = 19, 55.9%), 2 (N = 14, 41.2%), and 4 (N = 1, 2.9%).

**Table 2:** Location of tumors

Variable	Segmentectomy (n = 34)	Lobectomy (n = 107)	*P* value
Right upper lobe	8 (23.5%)	44 (41.1%)	.03
S1	3	16	
S2	3	18	
S3	2	10	
Right middle lobe	0 (0%)	7 (6.5%)	
Right lower lobe	12 (35.3%)	23 (21.5%)	
S6	7	6	
S7 or S8 or S9 or S10	5	17	
Left upper lobe	12 (35.3%)	19 (17.8%)	
S1+2 or S3	11	14	
S4 or S5	1	5	
Left lower lobe	2 (5.9%)	14 (13.1%)	
S6	0	6	
S8 or S9 or S10	2	8	

## Discussion

In recent years, segmentectomy has been increasingly performed for small peripheral non–small cell lung cancer with the aim of preserving pulmonary function. Randomized trials such as JCOG0802 and CALGB/Alliance 140503 have demonstrated smaller postoperative declines in %FEV1 after segmentectomy or sublobar resection compared with lobectomy^1^^,^^2^; however, these studies evaluated absolute changes and did not account for the number of resected segments.

In the present study, we evaluated predicted %FEV1 (and %VC for reference) values derived from Bolliger's segment-counting method after thoracoscopic pulmonary resections. First, we found that at 12 months after lobectomy, the measured %FEV1 was approximately 10% higher than the predicted value, whereas after segmentectomy the measured and predicted %FEV1 were almost identical, indicating good predictive accuracy. Similar discrepancies between measured and predicted postoperative pulmonary function after lobectomy have been reported previously.^5^ Second, when the functional loss was normalized per resected segment, the decrease in %FEV1 was significantly smaller after lobectomy than after segmentectomy (2.2% vs 4.6%). This segment count–based analysis is a key strength of our study and distinguishes it from previous reports.^E1^

The better baseline pulmonary function observed in the segmentectomy group likely reflects the higher proportion of female never-smokers with part-solid ground-glass nodules who underwent intentional segmentectomy and generally had well-preserved pulmonary function. Of note, our findings should not be interpreted as indicating that lobectomy is physiologically less invasive than segmentectomy. Parameters of spirometry, such as %FEV1 and %VC, do not fully capture the overall physiological burden associated with lobectomy. The higher mortality observed in the lobectomy group in the JCOG0802 trial^1^ suggests that lobectomy may impose physiological stress on cardiopulmonary reserve, exercise capacity, and other functional domains that are not adequately reflected by spirometry alone. Therefore, postoperative risk assessment should not rely solely on conventional pulmonary function tests.

A potential explanation for this finding is that postoperative functional recovery of the remaining ipsilateral lung may differ between segmentectomy and lobectomy. After lobectomy, the residual lobes can expand into the freed intrathoracic space, making measured function more likely to exceed predictions. Conversely, after segmentectomy, the preserved segments remain functional but may mechanically limit the expansion of other lobes. Thus, the compensatory mechanism observed after lobectomy may not occur to the same extent. Previous studies have reported particularly robust compensatory expansion after lower lobectomy, resulting in a larger discrepancy between measured and predicted values.^E2^ Another possible explanation is that intersegmental plane stapling may restrict expansion of the adjacent residual lung or alter regional compliance, resulting in greater functional loss per resected segment after segmentectomy than after lobectomy. However, this mechanism remains speculative. Previous studies have reported no significant differences in postoperative pulmonary function according to the method used for intersegmental plane division, including comparisons between stapler-based and energy device–based techniques.^E3^^,^^E4^ Therefore, the potential influence of intersegmental plane stapling should be interpreted cautiously and requires further investigation.

These findings suggest that the 19-segment method provides reasonable accuracy for thoracoscopic segmentectomy but tends to overestimate the decline in respiratory function after thoracoscopic lobectomy. These observations may have direct implications for preoperative risk stratification. Many institutions still depend on predicted postoperative %FEV1 calculated from segment-counting formulas to evaluate functional tolerability. If these formulas overestimate the functional loss associated with thoracoscopic lobectomy, some patients may be inappropriately categorized as high risk. Future prediction models should incorporate the type of resection and lobe-specific patterns of compensatory expansion, potentially supported by 3-dimensional imaging–based assessments of regional lung function, to more accurately estimate postoperative respiratory capacity in the era of minimally invasive surgery.

### Limitations

This study has limitations, primarily due to its small cohort. A statistically significant difference in tumor distribution between groups was observed (Table 2), which may have influenced the results. In addition, patient-specific variability in segmental volume was not accounted for. Also, most patients in our cohort had relatively well-preserved pulmonary function, and the number of patients with low preoperative %FEV1—who would most benefit from risk stratification—was insufficient to allow a meaningful subgroup analysis.

Despite these limitations, Bolliger's method remains widely used in clinical practice, and the observed segmental distribution and baseline characteristics likely reflect real-world clinical patterns. Nevertheless, our findings require validation in larger cohorts before they can be translated into clinical practice.

## Conclusions

In patients with generally preserved preoperative pulmonary function, Bolliger's method demonstrated good predictive accuracy after thoracoscopic segmentectomy. In lobectomy, however, the discrepancy persisted at 12 months, with measured %FEV1 being approximately 10% higher than predicted. Furthermore, the decrease in %FEV1 per resected segment was significantly smaller after lobectomy than after segmentectomy. These findings suggest that the conventional segment-counting method may overestimate postoperative respiratory decline after thoracoscopic lobectomy.

## Conflict of Interest Statement

The authors reported no conflicts of interest.

The *Journal* policy requires editors and reviewers to disclose conflicts of interest and to decline handling or reviewing manuscripts for which they may have a conflict of interest. The editors and reviewers of this article have no conflicts of interest.

## Figures and Tables

**Figure E1:**
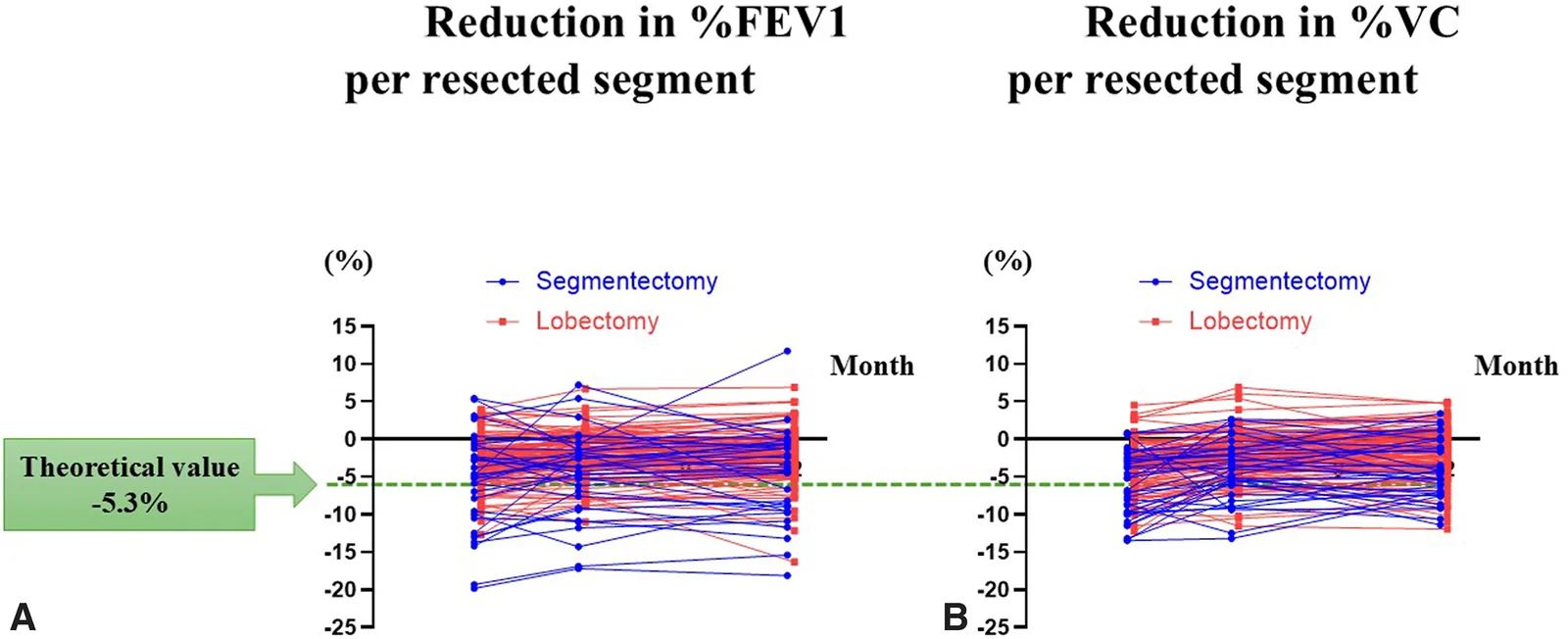
Reduction in %FEV1 (A) and %VC (B) per resected segment at 3, 6, and 12 months after surgery. Patients who underwent segmentectomy (*blue circles*) and lobectomy (*red squares*) are shown. Individual patient values are plotted, and lines connect serial measurements from the same patient over time. The *green dashed line* indicates the theoretical value (5.3%) calculated according to Bolliger's method. *%FEV1*, Percent forced expiratory volume in 1 second; *%VC*, percent vital capacity.
